# Biomarker Identification for Prostate Cancer and Lymph Node Metastasis from Microarray Data and Protein Interaction Network Using Gene Prioritization Method

**DOI:** 10.1100/2012/842727

**Published:** 2012-05-02

**Authors:** Carlos Roberto Arias, Hsiang-Yuan Yeh, Von-Wun Soo

**Affiliations:** ^1^Institute of Information Systems and Applications, National Tsing Hua University, Hsinchu 30013, Taiwan; ^2^Facultad de Ingeniería, Universidad Tecnológica Centroamericana, Tegucigalpa 11101, Honduras; ^3^Computer Science Department, National Tsing Hua University, Hsinchu 30013, Taiwan

## Abstract

Finding a genetic disease-related gene is not a trivial task. Therefore, computational methods are needed to present clues to the biomedical community to explore genes that are more likely to be related to a specific disease as biomarker. We present biomarker identification problem using gene prioritization method called gene prioritization from microarray data based on shortest paths, extended with structural and biological properties and edge flux using voting scheme (GP-MIDAS-VXEF). The method is based on finding relevant interactions on protein interaction networks, then scoring the genes using shortest paths and topological analysis, integrating the results using a voting scheme and a biological boosting. We applied two experiments, one is prostate primary and normal samples and the other is prostate primary tumor with and without lymph nodes metastasis. We used 137 truly prostate cancer genes as benchmark. In the first experiment, GP-MIDAS-VXEF outperforms all the other state-of-the-art methods in the benchmark by retrieving the truest related genes from the candidate set in the top 50 scores found. We applied the same technique to infer the significant biomarkers in prostate cancer with lymph nodes metastasis which is not established well.

## 1. Introduction

**Figure 1:**
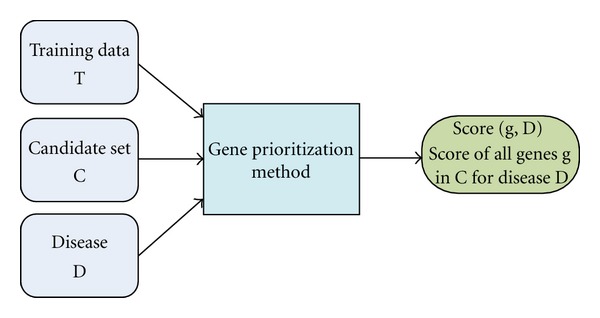
General gene prioritization overview.

Genetic diseases have been around for a long time. In the past they were just not understood or known. Nowadays we do have a better knowledge of the underlying mechanisms behind these diseases, for instance now it is understood that cancer is a mutated genetic disease [1] and many researchers in molecular genetics have identified a number of key genes and potential drug targets for various types of cancer [2]. Cancer is extremely complex and heterogeneous and it has been suggested that 5% to 10% of the human genes probably contribute to oncogenesis [3]. However, our current understanding is still limited, this is due to the very nature of the genetic mechanisms of life. It is not a trivial task to discover new genes involved with genetic diseases like cancer, as they usually do not work alone, but as a part of a mechanism inside the machinery of the cells. Current research in the discovery of new cancer related genes consists of several approaches, one direct approach called in vitro, and another one called “in-silico”. The in vitro approach is done by the biomedical community, they perform wet-lab experiments where they experiment with live tissue, comparing control and case cells. This approach is very accurate, but it is time consuming and extremely expensive, and sometimes it is not successful, since they might be investigating a gene that is not related with the disease. Here it is where bioinformatics provides tools to perform these studies in the so-called in silico environment. The bioinformatics studies are less accurate than in vitro ones, due to makeup of its source data that is usually noisy and incomplete [4]. On the other hand bioinformatics studies offer clues and hints to the biomedical researchers that help narrow the search for key genes and key mechanisms involved with a given disease, and it does it in a much cheaper fashion. Advances in this direction are essential for identifying new disease genes as biomarker in complex diseases. To achieve this goal our research is oriented to a line of bioinformatics investigation called whole genome “disease gene prioritization” (DGP). This line of research objective is to find disease-related genes, and to assign more relevant genes to the disease a higher score in such a way that higher scores have higher probability of being related to the disease in question. In general DGP is described in Figure 1, were it can be seen that DGP methods take as input training data, that is, information to indicate previous knowledge about the disease. Along with this data comes the candidate set, that represents the whole set of genes being studied, and that are going to be ranked or prioritized by the method. Finally the disease information is also input to the DGP method. Once the method has finished processing the input it will output the set of genes with a score, high scoring genes are believed to be relevant to the input disease.

In general there are two types of DGP, one of them is data and text-mining based, and the other is network based. Data and text-mining-based DGP methods rely on data mining techniques to mine disease relevant genes from literature or different bioinformatics sources like sequence information. Among these methods there are: the eVOC method that performs candidate gene selection based on the coocurrence of the disease name in PubMed abstracts using data mining methods [5], GeneSeeker that is a web-based tool that selects candidate genes of the disease under study based in gene expression and phenotypic data of human and mouse [6], the method proposed by Piro et al. [7] that uses spatial gene expression profiles and linkage analysis, disease gene prediction [8], and prospects [9] that use basic sequence information to classify genes as likely or unlikely to be involved with the given disease, SUSPECTS [10] an extended version of PROSPECTS that integrates annotation data from gene ontology (GO) [11], InterPro [12] and expression data. Along this line of research is also MedSim [13] that uses GO enrichment and their own similarities measures [14].

Additionally there are network based methods, these ones are based on network analysis tools applied on biological networks. Network methods have the advantage that there is an increasing availability of human protein interaction data, along with the maturity of network analysis. In the case of these kind of methods the training set is usually a set of genes that are called “seed genes,” these are genes that have been validated by wet lab experiments. Furthermore methods in this category can be classified in local and global methods, local methods use local information to the seeds, basically classifying by network proximity through the inspection of the seed genes or higher order neighbors in other words nodes in the network that are not directly adjacent to the seed nodes but are easily accessible by them. Global methods model the flow through the whole network to provide a score of the connectivity and impact of the seed genes. Either type, local or global, usually relies on the assumption that genes that are associated with diseases have a heavy interaction between each other [22]. The general idea behind network-based DGP is to assess how much genes interact together and how close they are to known diseases, integration of expression data from microarray into the network would improve its the accuracy, for more relevant biological information would be used. Among network-based methods: a method proposed by Chen et al. applies link-based strategies widely used in social and web network analysis such as Hits with Priors, PageRank and K-Step Markov to prioritize disease candidate genes based on protein interaction networks (PINs) [18]. Some various network-based approaches that predicted disease genes based on the protein network have obtained much better performance than traditional disease gene prediction approaches only based on the genome sequence alignment [23]. These kinds of the researches are associated with the long-held assumption that genes likely to interact directly or indirectly with each other are more likely to cause the same or similar diseases [24]. Wu et al. proposed a novel method, CIPHER (correlating protein interaction network and phenotype network to predict disease genes) [25] based on the phenotypic similarity and protein networks and they supposed that the phenotypic similarity among diseases can extract the disease-related genes on the network by measuring the direct neighbor (CIPHER-DN), shortest path (CIPHER-SP), or diffusion kernels. However, the direct neighbor strategy has some limitations to extract those indirect interaction genes and is more likely to be true for cases where two genes function in the same protein complex than in a pathway [26]. Shortest path analysis may yield a higher coverage and more novel predictions that are not so obvious to observe directly from the protein interaction data. The advantages of CIPHER perform genome-wide candidate gene prioritization for almost all human diseases but it does not work well for specific cancer due to not taking the relevant experimental data. They do not further select the active interaction relationships among protein while only a part of the interactions among a set of proteins may be active. These kinds of methods are inconsistent with previous studies which found that not all protein interactions occur at a specific condition [27]. More recently, Vavien is a system that uses the notion of topological profile to characterize a protein with respect to others [28]. Most of the aforementioned methods belong to the global or the local type of methods, integrating various sources of information to enrich their scoring. Another interesting fact is that as time passes the line the divides network based methods and data mining methods become less clear, this is due to the integration of data and text mining sources to network-based methods. This is the case of ENDEAVOR that takes a machine learning approach that builds a model with seed genes, and then that model is used to rank the candidate set according to a similarity score using multiple genomic data sources [17].

The accumulation of high-throughput data greatly promotes computational investigation of the expressions of thousands of genes and uses to manifest the expressions of genes under particular conditions. However, based on differential expression of the genes in the microarray data is likely to be incomplete, because there may be genes that are not differentially expressed but may be subtly involved in a pathway. The differential expression analysis only focuses on the selection of genes and does not pay attention to analyzing the interactions among them. Ma et al. proposes another method that performs the gene prioritization by Combining Gene expression and PIN (CGI) using Markov random field theory [19], CANDID that uses information from publications, protein domain descriptions, cross-species conservation measures, gene expression profiles and PIN to do a prioritization on the candidate genes that influence complex human traits [20], GeneRanks uses Google's PageRank algorithm and expression data to do gene prioritization [29], Mani et al. proposed a method called interactome dysregulation enrichment analysis (IDEA) to predict cancer related genes using interactome and microarray data [21], Karni et al. attempted to predict the causal gene from expression profile data and they identified a set of disease-related genes that could best explain the expression changes of the disease-related genes in terms of probable pathways leading from the causal to the affected genes in the network [30]. A summary table with the aforementioned methods can be found in Appendix D.

Scientific understanding of the biological mechanisms of cancer will help with the development of improved treatments for this disease, researchers around the world are attacking this issue with different approaches with a common goal: find a protocol to increase the probability of recovery from cancer. For this reason, our research is oriented towards this goal, and we have selected as our test domain prostate cancer. Motivated by the availability of rich information about this disease, and the fact that it is the third most common cause of death from cancer in male subjects according to the United States of America Library of Medicine.

In this paper we propose a method called gene prioritization based on microarray data with shortest paths, using voting extra scoring and edge flux strategy: (GP-MIDAS-VXEF), that is, a network-based DGP and a hybrid local and global method at it. It is based on the premise that disease relevant genes are on shortest paths involving seed genes like a local method. However uses the core of NetWalk to obtain disease relevant interactions making it also a global methods. Additionally boosting the scores using topological properties of the nodes that are considered to be “broker genes” as proposed by [31]. Finally is integrated differential expression data in the final score to increase biological meaning to the results.

This paper is organized as follows, following the introduction a brief background on graph theory is introduced, after which the Materials and Methods will follow where the source data and details on GP-MIDAS-VXEF will be presented. Then our results will be presented, finalizing this paper with the conclusions.

## 2. Graph Theory Background

 A graph is a data structure that represents a set of relationships between elements or objects. Formally a graph *G* is a pair defined by *G* = (*V*, *E*), where *V* is a set of elements that represent the nodes or vertices of the graph, the vertices in most applications hold the name of the attribute being represented. *E* is the set of edges, where each edge represents a relation between two vertices, an edge is defined by *E* = {(*u*, *v*) | *u*, *v* ∈ *V*}, which may hold additional information as weight, confidence or distance between nodes, therefore having *E* = {(*u*, *v*, *w*) | *u*, *v* ∈ *V*  and  *w* ∈ *Real*}. The edges may have directions, where (*u*, *v*)≠(*v*, *u*), in which case the graph is called directed graph, and when a direction is not important, the graph is called undirected graph.

### 2.1. Graph Properties

 Among the intrinsic properties of a graph there are: *Nodes*, the number of nodes in the network, formally *n* = |*V*|. *Edges*, the number of edges in the graph, formally *e* = |*E*|. *Graph Path*, is a sequence of vertices of the form {*v*_1_, *v*_2_, *v*_3_,…, *v*_*k*_} where *v*_1_ is the starting node and *v*_*k*_ is the destination node, and (*v*_*i*_, *v*_*i*+1_) ∈ *E*; the length of the path is defined by *l* = ∑_*i*=1_^*k*−1^ *w*_*i*_ where *w*_*i*_ ∈ (*v*_*i*_, *v*_*i*+1_, *w*_*i*_), when all weights are equal to 1 then the length of the path is *k* − 1. A shortest path from vertex *v* to *u* is one of the paths that have the least accumulated weight from *u* to *v*, note that there can be multiple shortest paths from one node to another.

### 2.2. Nodes Properties

 The most basic node property is the *degree* (*v*) that denotes the number of connections a node *v* has; in directed graphs there can be a distinction between incoming and outgoing connections, called *in-degree* and *out-degree*, respectively. Another property of the nodes is the *Clustering Coefficient*, this property measures which nodes in the network tend to cluster together and shows how close a node and its immediate neighbors are to become a full connected graph. Clustering coefficient is formally defined by (1) where ec(*v*) is the number of edges in the subgraph made only of node *v* and its neighbors, and nc(*v*) is the number of nodes of that graph:

(1) $$\begin{array}{c} \text{cc}\left(v\right)=\frac{2\text{ec}\left(v\right)}{\text{nc}\left(v\right)\left(\text{nc}\left(v\right)-1\right)}. \end{array}$$

Many other graph and node properties have been defined, a good reference for these properties can be found at [32].

## 3. Materials and Methods

### 3.1. Materials

 Current public PIN databases provide rich information and they mostly differ on the way they acquire or validate their data. For example, HPRD, BIND, MINT, and MIPS are manually curated. On the other hand, DIP and IntAct are based on literature mining and they achieve these using computational methods that retrieve the interaction knowledge automatically from published papers. Prieto and De Las Rivas have shown a limited intersection and overlap between the six major databases (BioGRID, BIND, MINT, HPRD, IntAct, DIP) [33]. The information contained in these databases is partly complementary and the knowledge of the protein interactions can be increased and improved by combining multiple databases.

To build more complete protein-protein interaction networks, we integrated PIN data warehouse included HPRD, DIP, BIND, IntAct, MIPS, MINT, and BioGrid databases (see Appendix C for details on these databases) which has successfully gathered 54,283 available and nonredundant human PIN pairs among 10,710 human related proteins into BioIR database [34]. This integration is a result of the availability of public protein interaction databases. Prostate cancer is a worldwide leading cancer and it is characterized by its aggressive metastasis. It is considered by the American Cancer Society as the second leading cause of cancer death among men, thus making this disease an important issue to study. However, up to date there are no reliable biomarkers can reliably associated with them. Understanding the differences in the biology of metastatic prostate cancer and non-metastatic primary tumors is essential for developing new prognostic markers and therapeutic targets. We used microarray data taken from [35] that consists of 62 primary tumors, 9 lymph nodes metastasis and 41 normal control samples. We applied those data into two groups, one is prostate primary tumor and normal samples and the other is prostate primary tumor with and without lymph nodes metastasis, respectively. We assign the weights to the protein networks for the edge flux step of our method and to boost the score in the final stage of the scoring phase. According to the genes from the microarray data, we extracted 8,123 genes and 43,468 protein-protein interactions to identify the prostate-related genes and subnetworks. These extracted genes are used as our training set for the prioritization process. The initial seed genes known to be related to the prostate cancer are extracted from public OMIM [36] database which stores gene-disease associations provided by summaries of publications and the list of the 15 seed genes are shown in Table 1, these genes are selected from [37] so that we can use a common ground for comparison. We took the KEGG pathway database [38] and PGDB database [39] that are manually curated database for prostate cancer and obtained 137 genes (Target Genes) as the truly disease-related genes for prostate cancer to compare the performance with the previous methods: CIPHER, Endeavour, HITS with priors, PageRank with priors, K-step Markov, and plain Random Walk with Restarts.

### 3.2. Methods

GP-MIDAS-VXEF is a hybrid local and global network based method for disease gene prioritization. The backbone of the method is based on the PIN, hence it relies on network analysis tools. The network analysis tools used along the method rest on the following well-documented assumptions:

- genes that have strong relationship between each other in the network tend to be closer together [22, 40, 41];
- important genes in the network show high degree and low clustering coefficient, since these genes are significant they are called in published literature “broker genes” [31].

**Figure 2:**
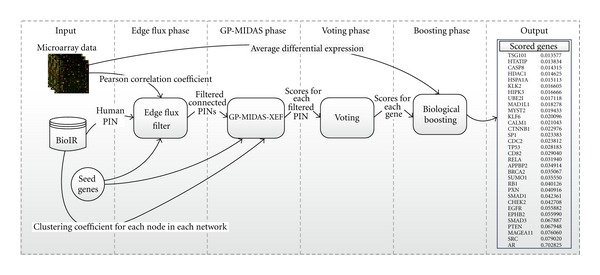
GP-MIDAS-VXEF workflow.

Although it is network based, it integrates expression data to find relevant interactions using a random walk with restarts (RWR) strategy, thus its global nature, after all, the RWR processes the network in its full extent (EF stage). Subsequently it does a shortest paths analysis along the networks that were generated by the EF stage. In each of those analysis it uses an extension of the basic scoring by incorporating a score boosting by means of considering the clustering coefficient of each of the genes in the network, covering the local nature. The previous two steps combined are called GP-MIDAS-XEF, GP-MIDAS for the basic shortest paths analysis, *X* for the extension using topological features, and EF for the incorporation of edge flux networks. Once the prioritization is done over all the EF networks, a set of scores is available for the voting phase, where the scores are integrated to produce a single-score base (voting stage). Finally, each gene score is boosted again using the average differential expression of each gene.  Figure 2 presents the general overview of GP-MIDAS-VXEF and how the input data is used on each of the stages. Following this introduction to the method, each of the stages are going to be presented.

#### 3.2.1. Edge Flux Filter

This is the first formal stage of the prioritization process, where the input PIN is analyzed using RWR with the purpose of finding relevant interactions to the specific domain under study. This model is applied using *γ* = 0.3 as probability of restart, as suggested by [22]. One disadvantage about the network based DGP is the use of noisy source data, therefore some steps are needed to filter out the source PINs in such a way that more relevant interactions are used in the core of the method. This is an open issue of research, nevertheless good results have been achieved like those of Komurov et al. that proposed a method called NetWalk [42]. This method is based on the execution of random walks on the network to obtain disease relevant interaction in the network. The steps that are executed during this stage are described in Algorithm 1.

Using the available microarray data the first step is to calculate the pearson correlation Coefficient (PCC) to determine the coexpressed relationship of the interactions in the PIN [43]. Two sets of PCC are calculated one for control sample (PCC^*N*^) and one for the case sample (PCC^*D*^). These Pearson correlation coefficients will be used as weights of the PIN that is input to the computation of the stochastic matrix

(2) $$\begin{array}{cc} & \text{Stochastic}\;{\text{Matrix}}_{ij}\\ & \;=\{\begin{array}{cc} 0, & \text{if}\;\text{there}\;\text{is}\;\text{no}\;\text{edge}\;\left(i,j\right),\\ \frac{\text{Weight}\left(i,j\right)}{\sum_{kadji}^{}\text{Weight}\left(i,k\right)}, & \text{otherwise}, \end{array} \end{array}$$

(3) $${ef}_{ij}={\text{RWR}}_{{\text{Scores}}_{i}}*{\text{StochasticMatrix}}_{ij},$$

(4) $$EF_{ij}=\log_{2}\left(\frac{ef_{ij}}{\text{ref}\_ef_{ij}}\right).$$

The main difference between Algorithm 1 and the one presented by [42] is that this method normalizes using weights from control expression data, and Komurov et al. use an unweighted network. The purpose of this normalization serves two purposes to unbias the results from structural bias that is natural in the RWR method and to unbias interactions that are similar between control and case samples. Notice that the resulting networks will no longer possess the *EF* values as weights but the weights of differential expression weights (diff_expr(*u*, *v*)) that are explained next, this sets the networks ready for shortest path analysis. Once this stage is over, the result is a set of networks that will be processed by the next phase.

To assign the expression weights to the EF-filtered PINA, the microarray data must be transformed in such a way that can be used to represent weights in the PIN, and that large weights indicate less interaction than small weights. This transformation has two steps, initially the values are updated using a sample of control expression microarray data, the effect of this operation is that values that are very similar between normal and cancer samples should have less impact on our analysis. To accomplish this we subtract the value from the cancer microarray data to the value of the control expression data as shown in (5), where there are *N* samples of control tissue and *M* samples of case tissue. The next step is to transform the values, the rationale behind this transformation is that expression values may be negative for underexpressed genes, and if these values are used as they are, our network may have negative weights, thus making shortest paths analysis more difficult. Equation (6) shows how the expression values are transformed:

(5) $$\begin{array}{cc} & \text{Expression}\;{\text{Value}}_{i}\\ & \;=\left|\frac{\sum_{n=1}^{N}\left(\text{control}\_{\text{expr}}_{n,i}\right)}{N}-\frac{\sum_{m=1}^{M}\left(\text{case}\_{\text{expr}}_{m,i}\right)}{M}\right|. \end{array}$$

(6) $$\begin{array}{cc} & \text{Transformed}\;\text{Expression}\;{\text{Value}}_{i}\\ & \;=-\ln\left(\frac{\left|\text{Expression}\;{\text{Value}}_{i}\right|-\min}{\max-\min}\right). \end{array}$$

Considering that the sign of the value in the microarray data represents over- or under-expression, and the fact that we want to make a representation of distance, for this is what we want in our quantitative analysis, we use the absolute value of the microarray data, then these results are normalized, using the max and min values found, by doing these two steps we get values in the range [0,1], where values closer to 1 mean that they are more expressed (either over expressed or under expressed). Finally we compute the negative of the natural logarithm on the previous results, this is to make smaller numbers (less expression level) become large distances, and bigger numbers (higher expression level) become short distances. The result of this step is a transformation of the gene expression, where more expressed genes have smaller value, and less expressed genes have higher values, in the next step we convert this values into distances between genes, thus more expressed genes relationships will become shorter distances than less expressed genes relationships. In the case the |Expression  Value_*i*_| = min we just set the whole result to be a big value, since in(0) is not defined. The result of this process is diff_expr(*u*, *v*), that represents the differential expression as a distance between nodes *u* and *v* in the PIN. Once the microarray expression data is transformed, it is ready to be integrated as weights into the PIN. Since we need the network to become a weighted one, where these weights are related to the specific interactions in disease-related network, we use the transformed values of the microarray data. However the microarray data provides transformed expression values for the genes, not for the relationship between genes. To overcome this issue, we combine the values of the two interacting genes together. For instance if we have microarray values {(SEPW1, 4.097), (BRCA1, 1.395), (AKT1, 2.006), (BACH1, 2.823), (AHNAK, 3.597)} and we have the following edges in our graph {(AKT1, AHNAK), (BACH1, BRCA1), (BRCA1, AKT1)}, then the first edge weight would be the addition of the transformed expression values of each of the vertices 2.006 + 3.597 = 5.603 providing the weight of the first edge. The resulting weighted edges of this instance would be {(AKT1, AHNAK, 5.603), (BACH1, BRCA1, 4.218), (BRCA1, AKT1, 3.401)}, this process results in all the relations in diff_expr(*u*, *v*) where (*u*, *v*) ∈ Interactions  of  *PIN*.

#### 3.2.2. Shortest Paths and Structural Prioritization: GP-MIDAS-XEF

At this phase each of the networks created by the NetWalk stage is going to be used as input of the GP-MIDAS-XEF. GP-MIDAS will do its prioritization based on the shortest paths, and then by boosting each gene score using the clustering coefficient of the gene in the specific network.

**Scoring of Genes with Shortest Paths.**

For this analysis all the shortest paths are computed, that is, for each pair of genes in the network the shortest paths are computed. As each of them is computed, the path is verified to check if any of the seed genes is on the resulting path, if so, these paths are added to the list of paths PathList to be considered in the scoring. Finally a score is computed for each gene.

**Compute the Score Function.**

Having all the paths stored in PathList we can compute the denominator denom using (7):

(7) $$\begin{array}{c} \text{denom}=\overset{n}{\underset{i=1}{\sum }}\frac{1}{l_{i}}, \end{array}$$

where *l*_*i*_ is the total length of the *i*th shortest path. Once the denominator is ready, we proceed to compute the score. For each gene *g* on the network we compute the score according to (8):

(8) $$\begin{array}{c} \text{Score}\left({\text{Gene}}_{i}\right)=\overset{\text{PathList}}{\underset{{\text{Gene}}_{i}\;\in \;{\text{Path}}_{j}}{\sum }}\frac{1/l_{j}}{\text{denom}}. \end{array}$$

The motivation behind (8) is that a gene that appears in more shortest paths or more times in the paths list is going to achieve higher score, the highest being 1 if the gene appears in all the found paths.

**Extending the Score of Genes.**

Cai et al. have demonstrated that disease genes in the network show particularly high degree and low clustering coefficient, defined in (1), they called this special genes *broker genes* [31]. Based on this idea, each of the previously computed scores are updated using (9). The boosting is computed from locally computed clustering coefficient of the node, and it affects that node alone:

(9) $$\begin{array}{cc} & \text{Score}\left({\text{Gene}}_{i}\right)\\ & \;=\text{Score}\left({\text{Gene}}_{i}\right)*\left(2-{\text{Clustering}\_\text{Coefficient}}_{\left({\text{Gene}}_{i}\right)}\right).\; \end{array}$$

By doing this boosting, genes with low clustering coefficient will have higher boosting, and high clustering coefficient will have lower boosting. The consequence is that disease-related genes are expected to have increased scores, a result that was achieved as will be demonstrated in the results section.

#### 3.2.3. Voting Phase

Since we are getting a set of thresholds *T* in the edge flux filtering phase to produce |*T*| different coexpressed networks. Those networks are built using the edges that have the values that result from the steps 8 to 14 in Algorithm 1, in other words the values on the ends of the two tails of the edge flux distribution. Second, we compute a score on each gene *g* of those networks and have a matrix of ranked genes where each row represents the position of the gene, as expressed in (10), where *S*_*i*_ is the score achieved by GP-MIDAS-XEF with threshold *i*. For all the tested ranked lists, we used rank aggregation to re-rank the genes. Borda count has been extensively studied which is originally a voting method based on positional-scoring rankings [44, 45]. We generate a weight vector w as follows: The top 1 ranked receive weight 1, top 2 ranked receive weight 1/2, by the same way to the weight 1/*k* for each last ranked (where *k* denotes the number of the genes in the network). This ranking is denoted in (11). Finally ScoreMatrix is summarized to provide a single score, this is done using (12) where pos_*i*_(*g*) denotes the position of gene *g* in the *i*th network:

(10) $$\begin{array}{c} \text{Score}\;\text{Matrix}=\text{EF}-\text{Phase}\_\text{Results}\_\text{Matrix}\\ \;\;=\langle S_{1},S_{2},\ldots ,S_{\left|T\right|}\rangle , \end{array}$$

(11) $$\begin{array}{c} \text{Score}\;{\text{Matrix}}_{i,j}=\frac{1}{j} \end{array},$$

(12) $$\begin{array}{c} \text{Voting}\;\text{Score}\left({\text{Gene}}_{g}\right)=\overset{\left|T\right|}{\underset{i=1}{\sum }}\left(\text{Score}\;{\text{Matrix}}_{i,{\text{pos}}_{i}(g)}\right). \end{array}$$

The rationale behind this equation is that a gene that appears more times in a higher position would get higher weight those genes that appear in lower positions. Finally, the final score of the genes is sum up the weights of the position of the genes from different coexpressed networks. The newly rank denotes the largest score wins higher positions from different network topology.

#### 3.2.4. Biological Boosting

 Before the voting phase we focus more on the gene prioritization based on the edge score in the network, however each gene in the candidate set will have a corresponding average differential expression (ADE) as defined by (5), where the average of the control expression and case expression samples are calculated and then substracted from each other. The rationale behind this computation is that larger values will indicate larger difference between disease tissue and normal tissue, and on the contrary values closer to zero will represent genes that their expression level does not change much between control and case samples. These values will serve in the last stage of the prioritization where the biological score boosting takes place, thus more boosting for higher differential expression and less boosting for lower differential expression.

Once the ADE is ready, the final stage of the prioritization takes in the single-score list from the Voting phase, this scores are boosted one last time using a normalized absolute differential expression (ADE) as was computed previously. The ADE values are normalized to ensure that the range of values are [0,1], numbers closer to 1 will represent numbers with higher difference between control and case samples, as opposed to values closer to zero. Equation (13) shows how the boosting is done. Notice that ADE in the boosting process uses only the gene specific expression data as a value to express how much important a node is in this analysis, unlike the diff_expr(*u*, *v*) that represented weights in the network:

(13) $$\begin{array}{cc} & \text{Final}\;\text{Score}\left({\text{Gene}}_{i}\right)\\ & \;=\text{Voting}\;\text{Score}\left({\text{Gene}}_{i}\right)*\left(1+\text{Normalized}\_{\text{ADE}}_{i}\right). \end{array}$$

## 4. Results and Discussion

### 4.1. Performance

The method was tested using Prostate Cancer as the domain for the experiments, as it is explained in the Materials section previously. The method is compared to HITS with Priors, K-step Markov and PageRank with Priors all from the ToppNet suite [18]; other methods in the benchmark are ENDEAVOR [17], CIPHER [25] and plain Random Walk with Restarts [46] using Pearson Correlation Coefficient for weights of the network. These methods were selected because they belong to the Network Based DGP methods class, and they do not integrate data and text mining capabilities in their prioritization. Seed Genes (Training Set) is not considered in the benchmark in any of the methods, therefore our method does include them in our benchmark. It is worth to mention that all 13 seed genes are recovered in the top 20 rank of the method. Another reason for the selection of these methods is their public availability, Vavien [28] is not considered because it can only handle 50 candidate genes, and the methods in the benchmark handle any amount of candidate genes.

**Figure 3:**
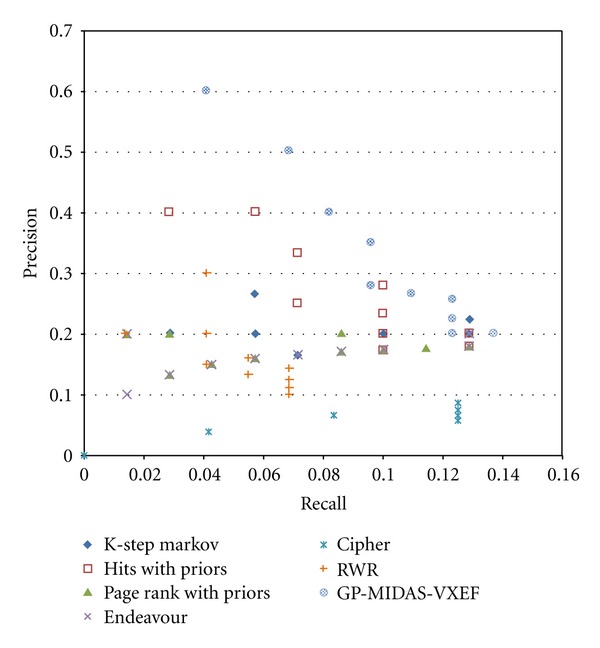
ROC curves comparing the performance of GP-MIDAS-VXEF with existent state-of-the-art network-based prioritization methods.

**Figure 4:**
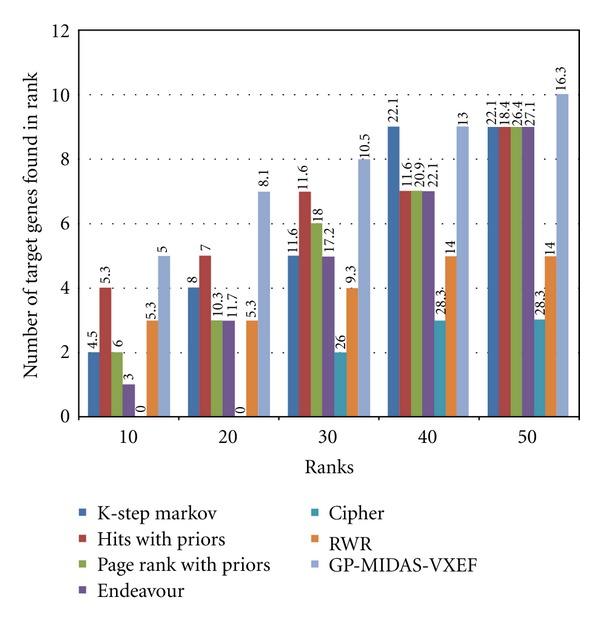
Target genes retrieved. Showing the amount of target genes retrieve on different ranks, on top of each bar the average position of the found genes is shown.

Figure 3 shows a precision-recall diagram were it is evident that our method has the best results among the rest of the methods in the benchmark. In Figure 4 absolute count of found genes per rank is presented. Additionally there is a number “Average Position” that represent the average position of the known cancer-related genes in the rank on the top of each bar. The figure clearly shows that GP-MIDAS-VXEF outperforms the other methods in the benchmark, and to resolve ties average position in the rank is also shown. For instance in the Top 40 ranks there is a tie between K-Step Markov and GP-MIDAS-VXEF, where both methods find 9 known cancer related gens. However GP-MIDAS-VXEF has an AP score of 13 which is less than the value of K-Step Markov with 22.11.

**Figure 5:**
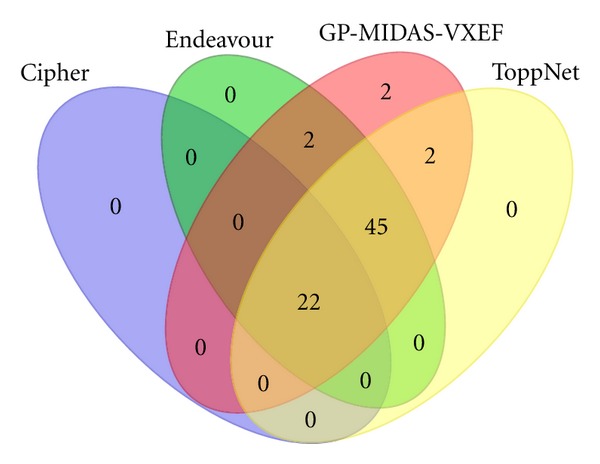
Venn diagram shows how the set of target genes is found amongst the different methods tested.

Additionally a Venn diagram is presented in Figure 5 where it is shown that most of the genes are found using ToppGene and GP-MIDAS-VXEF methods, where GP-MIDAS-VXEF outperforms all by finding two target genes that no other method finds. Furthermore, there are 22 overlapping genes showing that our method is consistent with previously found results.  Appendix A shows a list of target genes found by major methods.

### 4.2. Results Comparing Prostate Cancer and Normal Samples

**Figure 6:**
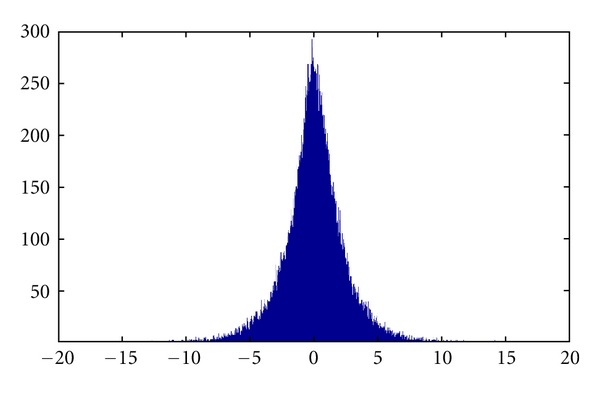
Edge flux values distribution.

**Figure 7:**
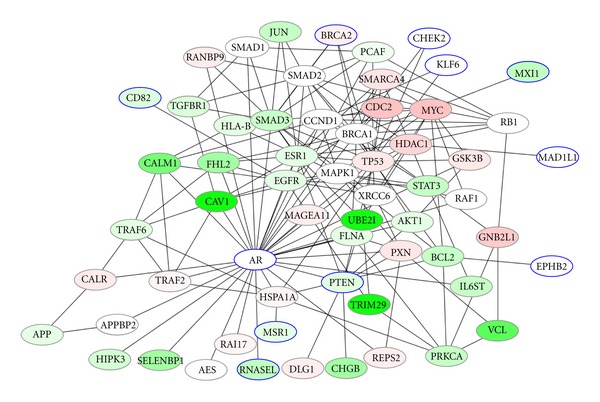
Prostate-normal experiment top 50 genes induced result network. Red color shows the higher difference in expression between prostate cancer and normal tissue sample, on the other hand the green color shows the smaller difference in expression between the samples. Nodes with node circle denote seed genes.

**Figure 8:**
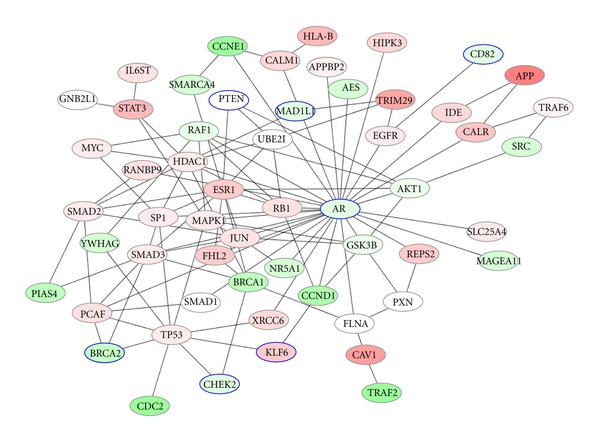
Prostate-metastatis experiment top 50 genes induced result network. Red color shows the higher difference in expression between prostate cancer and lymph node metastasis tissue sample, on the other hand the green color shows the smaller difference in expression between the samples. Nodes with node circle denote Seed Genes.

 We used the mean and variance to calculate the top 5% area as lower limits of the 95th percentile confidence interval with two tails in the distribution of the edge flux score which is shown in Figure 6. We present the two networks induced by top 50 genes from two kind of experiments which is shown in Figures 7 and 8. In Figure 7, we discovered that overexpressed gene androgen receptor (AR) being annotated in KEGG database as oncogene in the prostate cancer pathways also support the disease-related proteins in prostate cancer growth [23, 47]. BRCA1 and BRCA2 proteins play important role in DNA repair in both S and G2 checkpoint phase of the cell cycle and the results denote prostate cancer are strongly related to the tumor suppress genes (TP53, BRCA1, MYC, and PTEN) which have effect on the regulation of the cell cycle or promote apoptosis. Epidermal growth factor receptor (EGFR) family is also expressed in prostate cancer cells and their stimulation by EGF activates the mitogen-activated protein kinase (MAPK) and phosphatidylinositol-3 kinase (PI3K)/AKT pathways [48]. Those signal pathways stimulate cell cycle progression or survival which associated with cyclin D1 (CCND1) transcription and translation and the level of the BCL2. We found gene CALM1 that are associated with androgen receptor processes and interleukin 6 (IL 6) type of cytokine signaling pathways and their interactions with p38 MAPK may be the important factors related to the prostate cancer. Overexpression of MYC occurred frequently in most human prostate tumor databases revealed modules of human genes [49]. MXI1 protein associated with MYC which has also been suggested to play a role in prostate cancer [50–52]. Additionally we compare to previous results found in [53] that is focused on the construction of the regulatory network with emphasis in transcription factors. Transcription factors STAT3, MYC, and JUN are overlapped in both studies, providing some evidence to support the relationship of this genes to the prostate cancer. However our list does not overlap more since this study is not focussed in transcription factors only as in [53].

### 4.3. Results Comparing Prostate Cancer and Lymph Node Metastasis

 In Figure 8, Raf-1 kinase inhibitor protein (RKIP) was identified as the first physiologic inhibitor of the Raf/mitogen-activated protein kinase kinase/extracellular signal-regulated kinase (ERK) pathway [54]. Recently, RKIP has been recognized as a strong candidate for a metastasis suppressor gene in our experiments and we investigated RKIP expression is altered in clinical human lymph node metastases. Studies in cell cultures and animal models have suggested RKIP were found to be reduced or absent in metastatic variants of established cell lines derived from prostate cancer [54]. Androgen receptor coregulator, Filamin A (FlnA) is corresponded to hormone-dependence in prostate cancer and may be related to increased metastatic capacity [55]. We sought to determine FlnA expression across prostate cancer progression in human prostate cancer corresponded with metastatic potential. Histone deacetylase-1 (HDAC1) is association with SP1 was much weaker in lymph node metastatic than in nonmetastatic prostate cancer [56]. Our experimental data suggests induction of signalling activity via EGFR in prostate tumor cells and may provide a rationale for the use of EGFR inhibition in systemic prevention or treatment of lymph node metastatic [57]. In particular our experiments observed a properly designed inhibitor of nuclear receptor subfamily 5 (NR5A1) may be predicted to have therapeutic utility in the treatment of metastatic lymph node through suppression of androgen receptor. Previous studies have been studies that cyclin d1 (CCND1) is a mediator of prostate tumour cell proliferation and extend to lymph node metastasis [58]. V-src sarcoma viral oncogene homolog (SRC) has been specifically implicated in tumor growth and progression and resulting in both tumor growth and development of lymph node metastases [59]. This shows that targeting SRC family kinases may inhibit growth and lymph node metastases of prostate cancer. Not all biomarker genes found in lymph node metastasis (See Appendix B for details) can be explained at this moment. However our investigation shows that the molecular effects of lymph node metastasis related to AKT/GSK-3/AR signaling network along with the data presented above, that it may provide a biomarker indicative of prostate cancer with lymph node metastasis.

## 5. Conclusions

In this paper we present a method called GP-MIDAS-VXEF in which is successfully integrates several current state of the art acomplishments to achieve improved performance in the identification of disease-related genes. Through experimentation using Prostate Cancer as the domain, it has been shown that for the first top 50 genes GP-MIDAS-VXEF outperforms other methods, thus presenting an alternative in the gene prioritization field, that is, in terms of finding ranking known disease genes among the candidate gene set. The reason for our results are attributed to: the filtering phase where we obtain more relevant interactions, the combination of global and local network prioritization, using all-pairs shortest paths to find relevant routes for the seed genes, and for the particular boosting techniques that add structural and biological meaning to the results.

## Figures and Tables

**Figure 9:**
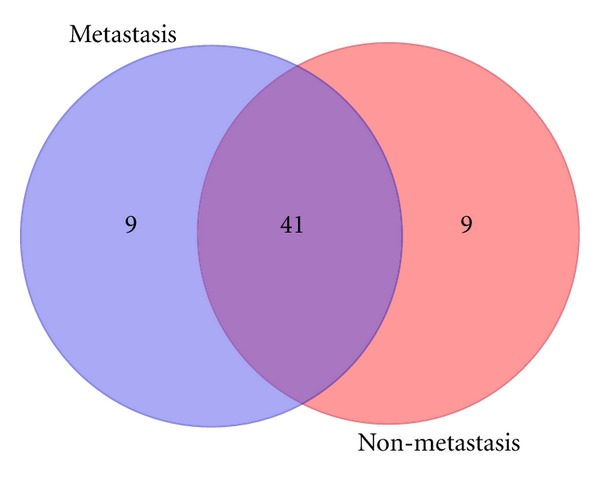
Venn diagram of top 50 genes.

**Algorithm 1:**
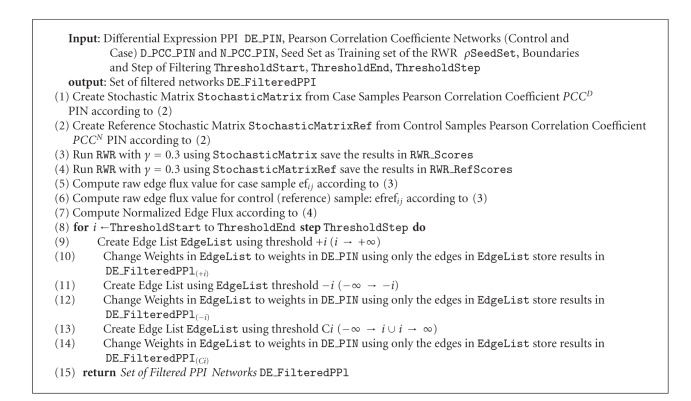
Overview of NetWalk Phase.

**Table 1:** Seed genes of prostate cancer from omim database.

Gene ID	Gene symbol	Gene name
367	AR	Androgen receptor
675	BRCA2	Breast cancer type 2 susceptibility protein
3732	CD82	CD82 antigen
11200	CHEK2	Serine/threonine-protein kinase Chk2
60528	ELAC2	Zinc phosphodiesterase ELAC protein 2
2048	EPHB2	Ephrin type-B receptor 2 precursor
3092	HIP1	Huntingtin-interacting protein 1
1316	KLF6	Kruppel-like factor 6
8379	MAD1L1	Mitotic spindle assembly checkpoint protein MAD
4481	MSR1	Macrophage scavenger receptor types I and II
4601	MXI1	MAX-interacting protein 1
7834	PCAP	Predisposing for prostate cancer
5728	PTEN/PTENP1	Phosphatidylinositol-3,4,5-trisphosphate 3-phosphatase, and dual-specificity protein phosphatase PTEN
6041	RNASEL	2–5A-dependent ribonuclease
5513	HPC1	Hereditary prostate cancer 1

**Table 2:** Target genes found across methods.

Method	Target genes
CIPHER	ATM, BRCA1, CAV1, CCND1, CDKN1A, CDKN1B, EGFR, EGR1, ESR1, ESR2, HIF1A, HRAS, MME, MSH2, MYC, NCOA3, NCOA4, PGR, RB1, RNF14, SMARCA4, TP53

ENDEAVOUR	ACPP, ANXA7, APC, ARMET, ATM, BCL2, BMP6, BRCA1, BTRC, CAV1, CCND1, CD44, CDH1, CDH13, CDKN1A, CDKN1B, CDKN2A, CTCF, CTNNA1, CTNNB1, CYP1B1, DAPK1, EDNRB, EGFR, EGR1, ERBB2, ERCC5, ESR1, ESR2, FAF1, FHIT, GGT1, GSTP1, HIF1A, HOXA13, HRAS, IGFBP3, IL12A, IL8, KLK10, KLK2, KLK3, MAP2K4, MME, MSH2, MYC, NAT1, NCOA3, NCOA4, NEFL, PGK1, PGR, PLAU, POLB, PTPN13, RARB, RASSF1, RB1, RNF14, SLC2A2, SMARCA4, SOX2, STMN1, TCEB1, TMEPAI, TNF, TP53, TYR, VDR

ToppNet (K-Step Markov, HITS with Priors, PageRank with Priors	ACPP, ANXA7, APC, ATM, BCL2, BMP6, BRCA1, BTRC, CAV1, CCND1, CD44, CDH1, CDH13, CDKN1A, CDKN1B, CDKN2A, CTCF, CTNNA1, CTNNB1, CYP1B1, DAPK1, EDNRB, EGFR, EGR1, ERBB2, ERCC5, ESR1, ESR2, FAF1, FHIT, GGT1, GSTP1, HIF1A, HOXA13, HRAS, IGFBP3, IL12A, IL8, KLK10, KLK2, KLK3, MAP2K4, MC1R, MME, MSH2, MYC, NAT1, NCOA3, NCOA4, NEFL, NME1, PGK1, PGR, PLAU, POLB, PTPN13, RARB, RASSF1, RB1, RNF14, SLC2A2, SMARCA4, SOX2, STMN1, TCEB1, TNF, TP53, TYR, VDR

GP-MIDAS-VXEF	ACPP, ANXA7, APC, ARMET, ATM, BCL2, BMP6, BRCA1, BTRC, CAV1, CCND1, CD44, CDH1, CDH13, CDKN1A, CDKN1B, CDKN2A, CTCF, CTNNA1, CTNNB1, CYP1B1, DAPK1, EDNRB, EGFR, EGR1, **EIF3S3**, ERBB2, ERCC5, ESR1, ESR2, FAF1, FHIT, GGT1, GSTP1, HIF1A, HOXA13, HRAS, IGFBP3, IL12A, IL8, KLK10, KLK2, KLK3, MAP2K4, MC1R, MME, MSH2, MYC, NAT1, NCOA3, NCOA4, NEFL, NME1, PGK1, PGR, PLAU, POLB, PTPN13, RARB, RASSF1, RB1, RNF14, SLC2A2, SMARCA4, SOX2, STMN1, TCEB1, TMEPAI, TNF, TP53, TYR, VDR, **VEGF**

**Table 3:** Top 50 Genes.

Rank	Using prostate and normal tissue	Using prostate and metastatis tissue
1	CAV1	CAV1
2	TP53	MAGEA11
3	MAGEA11	CALM1
4	CALM1	CALR
5	EGFR	TP53
6	UBE2I	FHL2
7	CALR	EGFR
8	SMAD3	APP
9	FHL2	JUN
10	HDAC1	SMAD3
11	APP	SMAD2
12	MYC	ESR1
13	JUN	RB1
14	ESR1	HIPK3
15	GNB2L1	BRCA1
16	HIPK3	SMAD1
17	SMAD2	GNB2L1
18	APPBP2	XRCC6
19	CDC2	UBE2I
20	BRCA1	HDAC1
21	RB1	CDC2
22	SMAD1	AES
23	PXN	STAT3
24	XRCC6	IL6ST
25	IL6ST	APPBP2
26	STAT3	PCAF
27	DLG1	REPS2
28	AES	FLNA
29	TRAF6	RAF1
30	FLNA	MYC
31	TRIM29	MAPK1
32	PCAF	TRAF6
33	REPS2	CCND1
34	AKT1	SMARCA4
35	PRKCA	HLA-B
36	RAF1	TRAF2
37	HLA-B	RANBP9
38	TRAF2	PIAS4
39	SMARCA4	GSK3B
40	MAPK1	TRIM29
41	CHGB	FOS
42	RANBP9	IDE
43	CCND1	SRC
44	GSK3B	PXN
45	HSPA1A	SLC25A4
46	BCL2	SP1
47	VCL	NR5A1
48	RAI17	YWHAG
49	TGFBR1	AKT1
50	SELENBP1	CCNE1

**Table 4:** Top 50 genes overlap.

Set	Total genes	Genes in set
Overlapped genes	41	HDAC1 IL6ST SMAD1 RB1 TRAF2 RAF1 BRCA1 APP CDC2 EGFR AKT1 FLNA AES SMAD2 REPS2 GSK3B SMARCA4 GNB2L1 STAT3 UBE2I TRAF6 MAPK1 MYC CAV1 JUN CCND1 RANBP9 HLA-B PCAF FHL2 TP53 TRIM29 CALR APPBP2 SMAD3 CALM1 MAGEA11 HIPK3 ESR1 PXN XRCC6

Genes in metastasis analysis	9	PIAS4 FOS YWHAG SLC25A4 SP1 SRC IDE CCNE1 NR5A1

Genes in nonmetastasis analysis	9	DLG1 VCL TGFBR1 CHGB SELENBP1 BCL2 PRKCA RAI17 HSPA1A

**Table 5:** Available biological networks sites.

Name	Acronym	URL
Human Protein Reference Database	HPRD	http://www.hprd.org/
Biomolecular Interaction Network Database	BIND	http://bond.unleashedinformatics.com/
Biological General Repository for Interaction Datasets	BioGRID	http://thebiogrid.org/
Database of Interacting Proteins	DIP	http://dip.doe-mbi.ucla.edu/
IntAct Molecular Interaction Database	IntAct	http://www.ebi.ac.uk/intact/
The MIPS Mammalian Protein-Protein Interaction Database	MIPS	http://mips.helmholtz-muenchen.de/proj/ppi/
Molecular Interaction Database	MINT	http://mint.bio.uniroma2.it/mint/
Kyoto Encyclopedia of Genes and Genomes	KEGG	http://www.genome.jp/kegg/
National Center for Biotechnology Information	NCBI	http://www.ncbi.nlm.nih.gov/

**Table 6:** Data and Text Mining Gene Prioritization Methods.

Method	Brief description	Reported results
Gene seeker	Gathers gene expression and phenotypic data from human and mouse from nine databases. Relies on the assumption that disease genes are likely to be expressed in tissues affected by that disease [6]	Offers a web-service to find disease-related genes to the input genetic localisation and phenotypic/expression terms

eVOC	Co-occurrence of disease name on PubMed Abstracts. It selects the disease genes according to expression profiles [5]	It was tested on 417 candidate genes, using 17 known disease genes. It successfully retrieved 15 of the 17 known disease genes and shrunk the candidate set by 63.3%

DPG	Basic Sequence Information [8].	They concluded that disease proteins tend to be long, conserved, phylogenetically extended, and without close paralogues.

Prospectr	Basic Sequence Information [10].	It achieved an enrichment of list of disease genes twofold 77% of the time, fivefold 37% of the time and twentyfold 11% of the time

Suspects	Extension of prospectr, incorporates GO [9, 15].	On average the target gene was on the top 31.23% of the resulting ranking list.

MedSim	GO enrichment and functional comparison [13].	It accomplished a performance of up to 0.90 in their ROC curve.

*Limitations*	Generally imposed by the source data which carries little knowledge about the disease. For instance GO terms include brief description of the corresponding biological function of the genes but only 60% of all human genes have associated GO terms, and they may be inconsistent due to differences in curators' judgement [16]	

**Table 7:** Network based gene prioritization methods.

Method	Brief description
Endeavor	Machine learning: using initial known disease genes; then multiple genomic data sources to rank [17]

HITS with priors Page rank K-Step markov	310 cm prioritization based on networks using social and web networks analysis [18]

CGI	Combination of protein interaction network and gene expression using markov random field theory [19]

CANDID	Uses publications, protein domain descriptions, cross species conservation measures, gene expression profiles and Protein Interaction Networks [20]

IDEA	Uses the interactome and microarray data [21]

*Limitations*	Most of these approaches include additional interactions predicted from coexpression, pathway, functional or literature data, but still fail to incorporate weights expressing the confidence on the evidence of the interactions. Another issue is that previous methods start with the given PIN without filtering its edges, to keep more relevant interactions to the disease

GP-MIDAS-VXEF	Our proposed method, integrates protein interaction network with normal and disease microarray data, using this integration we apply all-pairs shortest paths to find the significant networks and calculate the score for the genes. Additionally our method filters interactions, in such way the most relevant interactions are left for analysis

## Appendices

### A. List of Target Genes Found across Methods

Table 2 presents the list of target genes that were found in major gene prioritization methods. Notice that *EIF3S3* and *VEGF* are found only in GP-MIDAS-VXEF.

### B. Top 50 Genes from Prostate Cancer and Lymph Node Metastasis Extracted by Our Methods

Table 3 presents the list of biomarkers found using our methods. Additionally Table 4 shows the genes that are overlapped between the metastasis and nonmetastasis prioritization results, this is also shown graphically in Figure 9.

### C. Public Domain Protein Interaction Databases

Table 5 presents a list of publicly available biological networks databases.

### D. Disease Gene Prioritization Methods

Tables 6 and 7 show a summary on different disease gene prioritization methods.
